# Psychometric properties of an Arabic translation of the short form of the metacognition questionnaire (MCQ-30) in a non-clinical adult sample

**DOI:** 10.1186/s12888-023-05308-4

**Published:** 2023-10-31

**Authors:** Feten Fekih-Romdhane, Vanessa Azzi, Sahar Obeid, Sarah Gerges, Abir Sarray El Dine, Diana Malaeb, Michel Soufia, Souheil Hallit

**Affiliations:** 1 The Tunisian Center of Early Intervention in Psychosis, Department of Psychiatry “Ibn Omrane”, Razi Hospital, Manouba, 2010 Tunisia; 2 Faculty of Medicine of Tunis, Tunis El Manar University, Tunis, Tunisia; 3 School of Medicine and Medical Sciences, Holy Spirit University of Kaslik, P.O. Box 446, Jounieh, Lebanon; 4 Social and Education Sciences Department, School of Arts and Sciences, Lebanese American University, Jbeil, Lebanon; 5 Department of Biomedical Sciences, School of Arts and Sciences, Lebanese International University, Beirut, Lebanon; 6 College of Pharmacy, Gulf Medical University, Ajman, United Arab Emirates; 7 Applied Science Research Center, Applied Science Private University, Amman, Jordan; 8 Research Department, Psychiatric Hospital of the Cross, Jal Eddib, Lebanon

**Keywords:** Metacognition, MCQ-30, Arabic, Validation, Psychometric properties

## Abstract

**Background:**

Previous linguistic validations of the 30-item Metacognition Questionnaire (MCQ-30) have been performed in Western/Eastern populations, and no Arabic validated version exists to date for the wide Arabic-speaking populations in the Middle East-North African region and abroad. In this regard, we sought through the present study to test the psychometric properties of an Arabic translation of the MCQ-30 in a sample of Arabic-speaking community adults from Lebanon.

**Methods:**

The sample of this cross-sectional study consisted of 423 participants (mean age: 38.13 ± 11.03 years; 61.2% females). The Metacognition Questionnaire-short form, Teruel Orthorexia Scale and Emotion regulation questionnaire were used to assess metacognition, orthorexia nervosa and emotion regulation (cognitive reappraisal and expressive suppression) respectively.

**Results:**

Findings of Confirmatory Factor Analyses revealed that the five-factor model provided a good fit to the data. McDonald’s ω coefficients ranged from 0.78 to 0.94 for the five MCQ-30 subscales, and was of 0.93 for the total score, hence supporting the adequacy of scale reliability. Results also supported configural, metric, and scalar equivalence of the five-factor model across gender groups. The MCQ-30 subscales showed patterns of correlations with the emotion regulation and disordered eating constructs in the expected directions, providing evidence of the criterion-related validity of the measure. In particular, positive emotion regulation strategies (i.e., cognitive reappraisal) were negatively correlated with cognitive self-consciousness and need to control thoughts; whereas maladaptive emotion regulation strategies (i.e., expressive suppression) showed positive correlations with lack of cognitive confidence, negative beliefs and need to control thoughts. Additionally, all metacognition dimensions (except for cognitive self-consciousness) were significantly and positively correlated with higher levels of orthorexia nervosa behaviors.

**Conclusions:**

Our findings preliminarily suggest that the scale is valid, reliable, and can be recommended for use among the broad Arabic-speaking community worldwide.

## Introduction

Metacognition refers to psychological processes through which people consciously make efforts to appraise, modify or control their own cognition [1–5]. According to Lories and colleagues [6], metacognition is “a fundamental aspect of human cognition. Not only do we have cognitive activities but it would seem that they can apply to themselves: we have cognitions about cognition” (p.1). Metacognition may therefore be defined in a simpler way as ‘thinking about thinking’. Metacognition is thus a major component of human’s subjective experiences and behaviors [7]. Metacognition can either be adaptive (i.e. fostering metacognitive mode of functioning and successful problem solving when facing a problematic situation) or maladaptive (i.e., increased likelihood of choosing to respond to thoughts with repetitive negative thinking, and endorsing beliefs of the uncontrollability and danger of worry, which in turn cause and sustain emotional distress) [8, 9]. Accordingly, metacognition has been closely connected to multiple psychological and behavioral indicators. For instance, it has been demonstrated that adequate adaptive metacognitive skills positively affect students’ motivation and learning abilities (e.g., reading performance, learning strategies, academic self-efficacy, learning-related emotions) [10]. In addition, students with high adaptive metacognitive skills exhibit good academic performance [11] and more academic success [12]. Previous findings also indicated that several metacognitive experiences were positively and significantly linked to solving everyday problems and to a number of coping strategies (i.e., magical thinking, blaming others, and emotional outburst) [13]. It has also been argued that, when adaptive, metacognition is strongly related to emotional regulation, with ability to manage and regulate emotion increasing with the increase in metacognitive abilities [14, 15]. However, and following the metacognitive model of psychological disorder, metacognitive beliefs can drive maladaptive and prolonged patterns of thinking (such as persistent rumination or worry), which may, in turn, lead to maintained emotional distress [1, 16]. In this context, growing research pointed to greater levels of maladaptive metacognitive beliefs in various mental disorders, including depression [17], conversion disorder [18], personality disorders [19], addictive behaviors [20], bipolar disorder [21], psychotic disorders [22, 23], panic disorder [24], posttraumatic stress disorder [25], social anxiety [24], generalized anxiety disorder, obsessive-compulsive disorder, and eating disorders [26]. Furthermore, dysfunctional metacognitive beliefs have proven to significantly contribute to the activation and maintenance of maladaptive emotion regulation strategies [27]. Previous research showed that an individual’s capacity to regulate their own emotions closely depends on their metacognition abilities [28]. In particular, prior findings indicated that individuals with higher metacognition scores tend to have increased adaptive emotion-regulation strategies (i.e., cognitive reappraisal) [29]. Indeed, it has been suggested that, due to its cognitive rumination role, metacognition fulfills a fundamental function of emotion regulation and a significant contribution to emotional dysregulation [1]. As such, maladaptive metacognition beliefs have been recognized as a contributing factor for the development and maintenance of psychopathology following the self-regulatory executive function model [30]. The model suggests that, when not effectively regulated, repetitive and persistent thinking patterns may lead to psychological disorders [3, 31]. These patterns originate from a dysfunction in metacognitive beliefs about interpretations of mental events (e.g., beliefs about the positive and negative effects of rumination and worry) [31]. Consequently, increased attention was given to developing novel metacognition-based interventional approaches [32], which have proven effective in treating a range of mental disorders (e.g. [33–35],).

Valid and reliable measures are necessary for clinicians and researchers to assess metacognitive beliefs and their underlying theory across multiple populations. One widely used measure is the 30-item Metacognitions Questionnaire (MCQ-30) [2]. The MCQ-30 measures maladaptive metacognitive beliefs as traits that are involved in the choice of maladaptive coping strategies and, in turn, maintaining psychological dysfunction. It is a shortened and more convenient version of the parent instrument, the 65-item Metacognitions Questionnaire (MCQ), created by Cartwright-Hatton and Wells [36]. The MCQ-30 has a five-factor structure evaluating five metacognitive domains: (a) Positive beliefs about worry, which describes worrying as a problem-solving technique; (b) Cognitive self-consciousness, which evaluates the tendency to constantly focus attention on one’s own thinking processes; (c) (Lack of) Cognitive confidence, which concerns the efficacy of one’s own memory; (d) Negative beliefs, which describes worrying as being uncontrollable and dangerous; and (e) Need to control thoughts, which assesses the belief of the person about thoughts having to be controlled/suppressed. The MCQ-30 demonstrated adequate internal consistency, acceptable test-retest reliability, and satisfactory convergent validity as attested by positive correlations with indices of worry and obsessive–compulsive symptoms in a sample of students and university/health service employees aged 18 to 69 years [2]. In addition, the dimensional structure of the MCQ-30 was found to be invariant across gender [37, 38].

Since its development, the MCQ-30 has been translated and validated into multiple languages among community samples, including Spanish [37, 39], Italian [40], French [41], Serbian [42], Greek [43], Persian [44], Hindi (Indian) [45], Chinese [46], Korean [47], Malay [48], and Turkish [49, 50]. The MCQ-30 has also shown good psychometric properties in various clinical samples, such as patients with obsessive-compulsive disorder [51], anxiety and depressive disorders [39, 42], eating disorders [39], and epilepsy [52]. However, all these validations have been performed in Western/Eastern populations, and no Arabic validated version of the MCQ-30 exists to date for the wide Arabic-speaking populations in the Middle East-North African region and abroad. It is crucial to investigate whether the psychometric qualities of the original English version of the MCQ-30 could be replicated across cultures, in order to be used consistently worldwide. Cross-cultural validation studies would enable further evidence to be provided in support of the multicultural relevance of the metacognition construct in psychological practice, and gain more insight into specific targets for metacognitive therapy.

In this regard, we sought through the present study to test the psychometric properties of an Arabic translation of the MCQ-30 in a sample of Arabic-speaking community adults from Lebanon. To this end, we aimed to examine the factor structure, composite reliability, measurement invariance across gender groups, and concurrent validity of the Arabic MCQ-30 based on its correlations with measures of emotion regulation and disordered eating behaviors. We hypothesized that (1) Confirmatory Factor Analysis (CFA) would show that the theoretically assumed five-factor model will fit the data well (2) composite reliability of the five sub-scores and total MCQ-30 scores would be adequate, (3) subscales would be invariant by gender, and (4) scores would be significantly and positively correlated with measures of maladaptive emotion regulation strategies and orthorexia nervosa. These measures were included based on previous literature demonstrating significant correlations between dysfunctional metacognitive processes and maladaptive emotion regulation [27–29], as well as between maladaptive metacognitive beliefs and eating disorders, including orthorexia nervosa [26, 53].

## Methods

### Minimum sample size

A previous study suggested that the minimum sample size to conduct a confirmatory factor analysis ranges from 3 to 20 times the number of the scale’s variables [54]. Therefore, we assumed a minimum sample of 300 participants needed to have enough statistical power based on a ratio of 10 participants per one item of the scale, which was exceeded in this sample.

### Procedures

All data were collected via a Google Form link, between May and July 2021; Due to the coronavirus pandemic outbreak the data were gathered through snowball sampling method using an online questionnaire. The project was advertised on social media and included an estimated duration. Participants were included if they were adults (aged > 18 years) and were residents and citizens of Lebanon. We used the “duplicate data” option in Excel to make sure that no participant responded to the questionnaire more than once. All participants were asked to complete the anonymous survey questionnaire voluntarily after providing an informed consent. No compensation was offered. Instruments included in the questionnaire were presented in a pre-randomized order to control for order effects.

### Measures

The questionnaire was divided in three parts. In the first part, a written consent, confirming the approval of the participant to fill in the questionnaire was gathered. In the second part, respondents answered to questions assessing socio-demographic details (age, gender and education). In the last part of the study, participants completed a set of self-report measures as follows:

#### Metacognition questionnaire-short form (MCQ-30)

This tool [2] was used to assess dysfunctional metacognition processes on five dimensions, each composed of 6 items rated on a 4-point Likert scale from 1 (Disagree) to 4 (Agree): (a) Positive beliefs about worry (e.g., “l need to worry in order to remain organized”); (b) negative beliefs about uncontrollability and danger of worry (e.g., “l could make myself sick with worrying”); (c) (Lack of) cognitive confidence (e.g., “My memory can mislead me at times”); (d) Need to Control Thoughts (e.g., “I should be in control of my thoughts all of the time”); and (e) cognitive self-consciousness (e.g., “I monitor my thoughts”). Total scores were obtained by summing all items and ranged from 30 to 120, and each subscale score ranged from 6 to 24, with higher scores referring to greater levels of the corresponding dimension.

The MCQ-30 scale underwent two consequential translations. The procedure was initiated by a forward translation from English to Arabic, performed by a bilingual healthcare professional. Then, it was completed by a backward translation into English, performed by another bilingual primary care provider who was blinded to the scales’ notions and initial English versions. This process strictly follows international guidelines for a pertinent cultural adaptation of self-assessment scales [55]. No discrepancies were noted. At the end, the principal investigator, two psychiatrists and two psychologists revised and agreed to the measures’ final versions. A pilot study was conducted on 20 persons to make sure that all questions were well understood; no changes were done afterwards, therefore, these answers were kept in the database.

#### Teruel orthorexia scale

The Teruel Orthorexia Scale (TOS), validated in Lebanon [56, 57], is a 17-item instrument that assesses orthorexia nervosa with two separate dimensions [58]: 9 items for Healthy Orthorexia or “HeOr” (e.g., “I mainly eat foods that I consider healthy”) and 8 items for Orthorexia Nervosa or “OrNe” (e.g., “Thoughts about healthy eating do not let me concentrate on other tasks”). Responses are provided on a 4-point Likert-type scale ranging from 0 = strongly disagree to 3 = strongly agree. Scores by dimension were computed as the sum of the item responses. In this study, the internal consistencies were ω = 0.85 / α = 0.84 for the TOS OrNe and ω = 0.85 / α = 0.87 for the TOS HeOr. In this paper, the TOS OrNe score will be used.

#### Emotion regulation questionnaire (ERQ)

Validated in Lebanon [59], it is a ten-item scale, with questions scored on a 7-point Likert scale [60]. Two dimensions derive from this scale: Expressive Suppression (e.g. “When I want to feel more positive emotion (such as joy or amusement), I change what I’m thinking about”) and Cognitive Reappraisal (e.g. “I keep my emotions to myself”). Higher cognitive reappraisal scores are considered a positive emotion regulation strategy, whereas higher emotional suppression is considered as maladaptive emotion regulation strategies. In this study, the internal consistencies were ω = 0.81 / α = 0.84 for the cognitive reappraisal subscale and ω = 0.72 / α = 0.80 for the expressive suppression subscale.

### Statistical analyses

Since the data were collected using an online questionnaire, there were no missing values since responding to all questions was required. Using the SPSS AMOS software v.29, Confirmatory Factor Analyses (CFA) were conducted on the MCQ-30 scale based on its original factors solution. We used the maximum likelihood estimation. The χ^2^ to df ratio (χ^2^ /df), Comparative Fit Index (CFI), Tucker Lewis Index (TLI) and the Root Mean Square Error of Approximation (RMSEA) were used to evaluate the goodness-of-fit of the model [32]. The χ^2^ /df values of 2–5, RMSEA values ≤ 0.06 or TLI and CFI values ≥ 0.95 indicate a good-fitting model, whereas RMSEA values between 0.06 and 0.10 and TLI/CFI values ≥ 0.90 indicate acceptable fit. Furthermore, evidence of convergent validity was assessed in this sample using the average variance extracted (AVE), with values of ≥ 0.50 considered adequate [61].

**Gender invariance.** To examine gender invariance of MCQ scores, we conducted multi-group CFA [62] using the total sample. Measurement invariance was assessed at the configural, metric, and scalar levels [63]. Configural invariance implies that the latent MCQ variable(s) and the pattern of loadings of the latent variable(s) on indicators are similar across gender (i.e., the unconstrained latent model should fit the data well in both groups). Metric invariance implies that the magnitude of the loadings is similar across gender; this is tested by comparing two nested models consisting of a baseline model and an invariance model. Lastly, scalar invariance implies that both the item loadings and item intercepts are similar across gender and is examined using the same nested-model comparison strategy as with metric invariance [62]. Following previous recommendations [62, 64], we accepted ΔCFI ≤ 0.010 and ΔRMSEA ≤ 0.015 or ΔSRMR ≤ 0.010 as evidence of invariance. We aimed to test for gender differences on latent MCQ scores using an independent-sample *t*-test only if scalar or partial scalar invariance were established.

Composite reliability was assessed using McDonald’s ω and Cronbach’s α, with values greater than 0.70 reflecting adequate composite reliability [65]. To assess convergent and criterion-related validity, we examined bivariate correlations between MCQ scores and those on the additional measures included in the survey (ER and orthorexia nervosa) using the total sample. Based on Cohen’s recommendations [66], values ≤ 0.10 were considered weak, ~ 0.30 were considered moderate, and ~ 0.50 were considered strong correlations.

SPSS v.25 was used to compute bivariate analyses. Prior to the analyses, normality of distribution of the all continuous scales was confirmed via a calculation of the skewness and kurtosis as follows: TOS OrNe (S = 0.713; K = 0.154), cognitive confidence (S = 0.194; K = − 0.683), positive beliefs (S = 0.093; K = − 0.999), cognitive self-consciousness (S = − 0.286; K = − 0.032), negative beliefs (S = 0.299; K = − 0.861), need to control thoughts (S = 0.101; K = − 0.510), cognitive reappraisal (S = 1.127; K = 1.906), and expressive suppression (S = − 0.558; K = − 0.223). Values between − 2 and + 2 are considered acceptable to prove normal univariate distribution [67]. All scores were standardized before beginning the analysis. Pearson’s test was used to correlate the MCQ subscales scores with other continuous variables, whereas the Student’s t-test was used to compare two means. *P* < .05 was deemed statistically significant.

## Results

The sample consisted of 423 participants, with a mean age of 38.13 ± 11.03 years and 61.2% females. Other characteristics and description of the scores can be found in Table 1.

**Table 1:** Sociodemographic characteristics of the participants

Variable	Total (N = 423)	Males (N = 164)	Females (N = 259)
**Gender**			
Male	164 (38.8%)		
Female	259 (61.2%)		
**Education level**			
Secondary or less	54 (12.8%)	25 (15.2%)	29 (11.2%)
University	369 (87.2%)	139 (84.8%)	230 (88.8%)
	**Mean ± SD**	**Mean ± SD**	**Mean ± SD**
Age (in years)	38.13 ± 11.03 (min = 18; max = 75)	38.85 ± 11.33	37.68 ± 10.84
TOS Orthorexia nervosa	14.71 ± 4.98 (min = 8; max = 32)	14.95 ± 9.44	14.56 ± 5.00
MCQ30 total score	71.05 ± 15.90 (min = 35; max = 120)	72.16 ± 15.09	70.34 ± 16.38
MCQ30- Cognitive confidence	13.09 ± 4.57 (min = 6; max = 24)	13.32 ± 4.44	12.95 ± 4.65
MCQ30- Positive beliefs	13.57 ± 5.32 (min = 6; max = 24)	14.05 ± 5.18	13.27 ± 5.39
MCQ30- Cognitive self-consciousness	17.49 ± 3.91 (min = 6; max = 24)	16.88 ± 4.15	17.87 ± 3.70
MCQ30- Negative beliefs	13.13 ± 5.04 (min = 6; max = 24)	13.48 ± 4.77	12.92 ± 5.20
MCQ30- Need to control thoughts	13.76 ± 4.26 (min = 6; max = 24)	14.42 ± 3.87	13.34 ± 4.45
Expressive suppression	32.92 ± 20.84 (min = 4; max = 28)	15.82 ± 6.65	15.40 ± 6.72
Cognitive reappraisal	5.89 ± 4.70 (min = 6; max = 42)	20.02 ± 5.18	18.39 ± 5.74

TOS = Teruel Orthorexia Scale; MCQ30 = Metacognition Questionnaire 30 items

### Confirmatory factor analyses of the MCQ-30 scales

CFA results indicated that the fit indices of the original five-factor model [2] were adequate: χ^2^/df = 1168.84/395 = 2.96, RMSEA = 0.068 (90% CI 0.064, 0.073), SRMR = 0.074, CFI = 0.902, TLI = 0.893. When adding a correlation between residuals of items 21 and 22, the fit indices improved as follows: χ2/df = 1105.05/394 = 2.80, RMSEA = 0.065 (90% CI 0.061, 0.070), SRMR = 0.074, CFI = 0.910, TLI = 0.901. The standardized estimates of factor loadings were all adequate (see Table 2). The CFA results of the second-order CFA results were good as follows: χ^2^/df = 1105.06/394 = 2.81, RMSEA = 0.065 (90% CI 0.061, 0.070), SRMR = 0.075, CFI = 0.910, TLI = 0.901. The convergent validity was adequate for all subscales as shown by adequate AVE values, except for the need to control thoughts as follows: cognitive confidence (= 0.57), positive beliefs (= 0.72), cognitive self-consciousness (= 0.54), negative beliefs (= 0.61), need to control thoughts (= 0.42), and total model (= 0.64). The correlations between factors were as follows: 1–2 (*r* = .41), 1–3 (*r* = − .07), 1–4 (*r* = .49), 1–5 (*r* = .44), 2–3 (*r* = .15), 2–4 (*r* = .49), 2–5 (*r* = .49), 3–4 (*r* = .10), 3–5 (*r* = .12), and 4–5 (*r* = .80).

We also calculated the fit indices for the one-factor model; the results were not good: χ^2^/df = 4469.91/405 = 11.53, RMSEA = 0.158 (90% CI 0.154, 0.162), SRMR = 0.147, CFI = 0.462, TLI = 0.422.

**Table 2:** Items of the metacognition scale- 30 items (MCQ30) in English and Factor Loadings Derived from the Confirmatory Factor Analysis (CFA) in the total sample

	Total
**Factor 1 : MCQ30- Cognitive confidence**	
1. I have little confidence in my memory for words and names	0.68
2. My memory can mislead me at times	0.74
3. I have a poor memory	0.82
4. I have little confidence in my memory for places	0.71
5. I do not trust my memory	0.80
6. I have little confidence in my memory for actions	0.77
**Factor 2 : MCQ30- Positive beliefs**	
7. Worrying helps me to avoid problems in the future	0.75
8. I need to worry in order to remain organised	0.85
9. Worrying helps me to get things sorted out in my mind	0.88
10. Worrying helps me cope	0.90
11. Worrying helps me to solve problems	0.90
12. I need to worry to work well	0.81
**Factor 3 : MCQ30- Cognitive self-consciousness**	
13. I think a lot about my thoughts	0.40
14. I am aware of the way my mind works when I am thinking through a problem	0.73
15. I monitor my thoughts	0.80
16. I am constantly aware of my thinking	0.82
17. I pay close attention to the way my mind works	0.81
18. I constantly examine my thoughts	0.76
**Factor 4 : MCQ30- Negative beliefs**	
19. My worrying is dangerous for me	0.75
20. I could make myself sick with worrying	0.81
21. My worrying thoughts persist, no matter how I try to stop them	0.79
22. I cannot ignore my worrying thoughts	0.74
23. My worrying could make me go mad	0.81
24. When I start worrying I cannot stop	0.80
**Factor 5 : MCQ30- Need to control thoughts**	
25. If I did not control a worrying thought and the nit happened, it would be my fault	0.74
26. I should be in control of my thoughts all of the time	0.50
27. Not being able to control my thoughts is a sign of weakness	0.63
28. I will be punished for not controlling certain thoughts	0.76
29. It is bad to think certain thoughts	0.65
30. If I could not control my thoughts, I would not be able to function	0.59

### Gender invariance

As reported in Table 3, all indices suggested that configural, metric, and scalar invariance was supported across gender for the 5-factor model and the second-order model. The results showed that there was no statistically significant difference between males and females in all metacognition dimensions, except for the cognitive self-consciousness where females scored significantly higher than males (Table 4).

### Composite reliability

Composite reliability of scores was adequate in the total sample for the total scale (ω = 0.93 / α = 0.92), cognitive confidence (ω = 0.86 / α = 0.89), positive beliefs (ω = 0.94 / α = 0.94), cognitive self-consciousness (ω = 0.84 / α = 0.86), negative beliefs (ω = 0.89 / α = 0.91), need to control thoughts (ω = 0.78 / α = 0.82).

**Table 3:** Measurement Invariance of the meta-cognition scale (MCQ-30) across gender in the total sample

Model	χ²	*df*	CFI	RMSEA	SRMR	Model Comparison	Δχ²	Δ*df*	*p*	ΔCFI	ΔRMSEA	ΔSRMR	
Model 1 : First order model													
Males	809.45	394	0.855	0.080	0.090								
Females	877.24	394	0.908	0.069	0.078								
Configural	1754.51	790	0.881	0.054	0.089								
Metric	1774.10	815	0.882	0.053	0.091	Configural vs. metric	19.59	25	0.767	0.001	0.001	0.002	
Scalar	1795.59	840	0.882	0.052	0.091	Metric vs. scalar	21.49	25	0.664	< 0.001	0.001	< 0.001	
Model 2 : Second-order model													
Males	823.32	399	0.852	0.081	0.098								
Females	891.88	399	0.906	0.069	0.085								
Configural	1715.56	798	0.887	0.052	0.098								
Metric	1735.28	823	0.888	0.051	0.099	Configural vs. metric	19.72	25	0.761	0.001	0.001	0.001	
Scalar	1767.48	852	0.887	0.051	0.100	Metric vs. scalar	32.2	29	0.311	0.001	< 0.001	0.001	

*Note.* CFI = Comparative fit index; RMSEA = Steiger-Lind root mean square error of approximation; SRMR = Standardized root mean square residual

**Table 4:** Comparison between sexes in terms of the metacognition 30 items scale and subscales scores in the total sample

	MCQ-30 total score	MCQ30- Cognitive confidence	MCQ30- Positive beliefs	MCQ30- Cognitive self-consciousness	MCQ30- Negative beliefs	MCQ30- Need to control thoughts
Males	74.69 (17.16)	14.76 (3.90)	15.17 (5.80)	15.48 (4.72)	14.20 (4.58)	15.09 (4.26)
Females	76.71 (16.29)	14.38 (4.76)	16.02 (5.16)	17.37 (3.88)	14.51 (5.05)	14.43 (3.88)
*t*	1.219	0.892	1.582	4.309	0.633	1.634
*p*	0.223	0.373	0.114	**< 0.001**	0.527	0.103

Numbers in bold indicate significant p-values. Numbers are shown as mean (SD)

### Validity

Higher cognitive-confidence was weakly and significantly associated with higher orthorexia nervosa (*r* = .21; *p* < .001) and expressive suppression (*r* = .23; *p* < .001). Higher positive beliefs were also associated higher orthorexia nervosa (*r* = .25; *p* < .001) and expressive suppression (*r* = .18; *p* < .001). Higher cognitive self-consciousness was significantly and weakly associated with lower cognitive reappraisal (*r* = − .15; *p* < .001). Higher negative beliefs were significantly and weakly associated with more expressive suppression (*r* = .20; *p* < .001) but negatively associated with age (*r* = − .11; *p* < .001). Finally, higher need to control thoughts significantly and weakly associated with more expressive suppression (*r* = .24; *p* < .001) but negatively associated with age (*r* = − .11; *p* < .001) (Table 5).

**Table 5:** Correlation matrix of continuous variables

Variable	1	2	3	4	5	6	7	8	9
1. MCQ30- total score	1								
2. MCQ30- Cognitive confidence	0.65***	1							
3. MCQ30- Positive beliefs	0.75***	0.39***	1						
4. MCQ30- Cognitive self-consciousness	0.40***	− 0.03	0.19***	1					
5. MCQ30- Negative beliefs	0.81***	0.43***	0.46***	0.17**	1				
6. MCQ30- Need to control thoughts	0.77***	0.36***	0.43***	0.18***	0.66***.	1			
7. Orthorexia nervosa	0.31***	0.21***	0.25***	0.01	0.29***	0.26***	1		
8. Cognitive reappraisal	− 0.07	− 0.01	− 0.04	− 0.15**	0.02	− 0.07	− 0.03	1	
9. Expressive suppression	0.25***	0.23***	0.18***	− 0.03	0.20***	0.24***	0.18***	− 0.45***	1
10. Age	− 0.09	0.04	− 0.08	− 0.04	− 0.11*	− 0.11*	0.06	− 0.04	− 0.001

Numbers refer to Pearson correlation coefficients. *p < .05; **p < .01; ***p < .001

## Discussion

The goal of this study was to investigate the psychometric characteristics of the Arabic version of the MCQ-30 in terms of factor structure, internal consistency, gender invariance, and validity. Findings provided support for the good psychometric properties of this version. More specifically, findings of CFA revealed that the five-factor model provided a good fit to the data. Results also established sufficient evidence of good reliability and measurement invariance, as well as acceptable criterion-related validity. These findings suggest that the scale can be recommended for use among the broad Arabic-speaking community worldwide.

As expected, the present findings replicated the originally proposed five-factor solution of metacognitive beliefs and related processes: positive beliefs about worry, cognitive confidence, beliefs about the need to control one’s thoughts, negative beliefs about the dangers and uncontrollability of worrying, and cognitive self-consciousness. This five-factor structure is similar to that of the full form MCQ [36], the English version of the MCQ-30 [2], as well as later validations in various populations and countries (e.g., Turkey [49, 50], Spain [37], UK [68], France and Belgium [41], Italy [40], Greece [43], South Korea [47], and China [46]). Covering all the five conceptually distinct dimensions in clinical research and practice could be more informative than computing a single total score, and may help provide comprehensive assessments of the multidimensional complex construct of metacognition. It is of note that a correlation was added between items 21 “My worrying thoughts persist, no matter how I try to stop them” and 22 “I cannot ignore my worrying thoughts”; this is not surprising since both items tackle the same concept (worrying thoughts) and had high collinearity. Furthermore, findings from our sample revealed that McDonald’s ω coefficients ranged from 0.78 to 0.94 for the five MCQ-30 subscales, and was of 0.93 for the total score, hence supporting the adequacy of scale reliability. McDonald’s ω was selected as a measure of composite reliability in this study because of known problems with the use of Cronbach’s α [69]. It is of note, however, that the reliability of the MCQ-30 was checked in the vast majority of linguistic validation studies using Cronbach’s α (e.g., α values for both the total and all five subscales ranged between 0.79 and 0.85 [41], 0.71-0.89 [47], 0.75-0.92 [46], and 0.69-0.89 [37] for the French, Korean, Chinese, and Spanish versions, respectively).

Besides examining the factor structure and internal consistency, we investigated the invariance of the Arabic MCQ-30 across gender. Results supported configural, metric, and scalar equivalence of the five-factor model across gender groups. Previous studies (e.g. [37, 38]) also confirmed the assumption of measurement invariance for the five-factor structure of the MCQ-30 across gender. Obtaining evidence of gender-related invariance allows for making statistically meaningful comparisons between males and females. It is of note that the model fit worsened from the overall model conducted on the whole sample to the configural model for the two gender subsamples; this indicates that there are some slight differences in how males and females see the latent structure. This might be explained that some items are an indicator of a different latent variable in males and females. When testing models separately for males and females, the model is adequate for females but is suboptimal in males. However, we should keep in mind that the number of males in the total sample is small, which might have caused this discrepancy. In the present study, only cognitive self-consciousness dimension showed statistical significant differences by gender, with female respondents reporting higher scores compared to males. Cognitive self-consciousness is referred to as the individual’s preoccupation with own thoughts and self-monitoring of the beliefs (e.g., ‘‘I pay close attention to the way my mind works”). A close look at the previous literature indicates wide variations in the findings related to gender differences in this metacognitive dimension. The original validation study of the MCQ found that healthy women displayed significantly lower scores in the subscale ‘cognitive self-consciousness’ compared to healthy men [36]. Later, the initial development and validation study of the MCQ-30 showed lower (though non-significant) scores for healthy females in the subscale ‘cognitive self-consciousness’ and in overall scores compared to males [2]. Another study by Welsh et al. [70] revealed similar findings. In contrast, results from other studies using the MCQ-30 either concurred with our findings that females reported higher scores [71], or observed no significant gender differences in this subscale [37, 72]. These mixed findings might be due to differences in methodology and samples of these studies, and call for additional studies to test the invariance of the factor model of the MCQ-30 in different populations and cultures according to gender.

The MCQ-30 subscales showed patterns of correlations with the emotion regulation construct in the expected directions, providing evidence of the criterion-related validity of the measure and the metacognitive theory suggesting that beliefs about internal states may influence the use of unhelpful strategies. In particular, positive emotion regulation strategies (i.e., cognitive reappraisal) were negatively correlated with cognitive self-consciousness; whereas maladaptive emotion regulation strategies (i.e., expressive suppression) showed positive correlations with lack of cognitive confidence, negative beliefs and need to control thoughts. These findings are consistent with earlier research that indicated that metacognitive beliefs relating to various mental states including emotions could be determinant in the ability to regulate emotions [73]. Metacognitive abilities are closely associated with enhanced adaptive emotion regulation abilities [14, 15]. Additionally, all metacognition dimensions (except for cognitive self-consciousness) were significantly and positively correlated with higher levels of orthorexia nervosa behaviors. Research found that metacognitive beliefs seem to be implicated in a range of disordered eating patterns and behaviors and overrepresented in people with eating disorders diagnoses and eating problems compared to the general population (for review, see [39]). Interestingly, and in agreement with the current findings, prior evidence suggests that components of metacognition might differently predict psychopathological symptoms [47]. Beyond providing support for the validity of the scale, our findings contribute to the increasing amount of available data by reinforcing the importance of assessing metacognition in clinical practice and research.

Finally, another interesting finding of our study was that higher MCQ-30 scores were inversely correlated with age in our adult sample. Studies having assessed metacognition using the MCQ-30 demonstrated that age seem to influence the expression of metacognitive beliefs. For instance, and in agreement with our findings, a study among UK adults from the general population revealed that older participants scored significantly lower on all MCQ-30 factors (except for cognitive confidence) [68]. Likewise, a study among Turkish university students demonstrated that age correlated negatively with need to control thoughts, cognitive self-consciousness, and uncontrollability/danger subscale scores, as well as with the total MCQ-30 score [74]. Another study in a clinical population reported a negative correlation between age and metacognitive beliefs about the need to control thoughts [51]. One plausible explanation can be that greater life experience leads to greater metacognitive efficiency and more accurate self-knowledge, thus suggesting that older adults tend to adopt maladaptive metacognitive beliefs to a lesser extent compared to younger adults.

### Limitations and future research perspectives

Certain limitations need to be addressed in future research. First, the study had a cross-sectional design and relied on self-report measures. Second, some important psychometric characteristics (such as test-retest reliability) have not been investigated in the context of this study. Future validation studies of the Arabic MCQ-30 are required to ensure the consistency of the scale over time and across investigators. Third, a web-based and convenience sampling method were used; which may limit the representativeness of our sample. Caution should be advised while interpreting the results of the configural model since the fit indices are slightly above the cutoff values for model fit adequacy. Finally, adults from a single Arab country were included; which precludes any generalization of the results to other Arab populations and contexts, especially since Lebanese culture and environment may differ from other Arab countries. Further studies in Arabic-speaking samples with greater cultural diversity are needed to examine if the factorial model of the Arabic MCQ-30 evidences cross-country and cross-cultural measurement invariance.

## Conclusion

The present findings provide support for the reliability and the validity of the five-factor model of the Arabic version of the MCQ-30 consistently in both genders. Although future studies still need to investigate whether the MCQ-30 performs similarly across diverse Arab cultures/countries; we preliminarily suggest that the Arabic version can be recommended for use to assess metacognitive beliefs among Arabic-speaking populations.

## Data Availability

All data generated or analyzed during this study are not publicly available due to restrictions imposed by the ethics committee. The dataset supporting the conclusions is available upon request to the corresponding author (SH).
